# New Appliances and Things Medical

**Published:** 1899-09-09

**Authors:** 


### NEW APPLIANCES AND THINGS MEDICAL.

**[We shall be glad to receive, at our Office, 28 & 29, Southampton Street, Strand, London, W.C., from the manufacturers, specimens of all new preparations and appliances which may be brought out from time to time.]**

### WIESBADEN KOCHBRUNNEN SALT, WATER, AND LOZENGES.

(London Agency: L. R. Voigt and Co., 25, Great Tower Street, E.C.)

The Kochbrunnen salt is obtained by evaporating water obtained from the famous Wiesbaden spring. It is intended for internal use, and by dissolving one teaspoonful of the salt in a tumblerful of hot water a corresponding dose of the original spring water is obtained. As is well known, the contained salt acts as a mild cathartic without producing any irritation of the mucous membrane of the alimentary tract. It promotes digestion, and causes an active secretion of gastric juice, lessening the tenacity of the mucus in the stomach, intestines, bladder, and respiratory organs. The lozenges are composed of the same salts, and the quantity contained in two of them equals that which is contained in a tumblerful of the natural water. They may be taken in the ordinary way of other lozenges, or dissolved first in water. They should be most useful for travellers. The Wiesbaden salts dissolved in water constitute an effectual and efficacious external application for various gouty and catarrhal conditions of the mucous membranes which can be reached by such applications; for nasal, pharyngeal, laryngeal, and gastric catarrh, when employed in the form of the spray or douche, they have frequently been of the greatest value.

### NURSES' CHART.

Messrs. J. Wright and Co., of Bristol, have sent us a sample of a new nurses' chart which they have published. It is about the size of an ordinary temperature chart, and is so ruled as to facilitate the recording of the various events which happen in the course of the day's nursing, the various observations which the nurse has to make, and the amounts of food given from time to time. There can be no doubt that such a tabulated record will convey information to the physician much more readily, and with much less chance of error, than the written reports which some nurses present to us on entering the sick-room. These charts will also serve a good purpose in reminding the nurse herself how the feeding is going on, and there is no doubt that they will be of very great service whenever a fresh nurse comes on duty, and will tend much to promote continuity of treatment. It should be mentioned that at the back of the chart is a list of all sorts of articles of diet, by aid of which the doctor may jog his memory in suggesting a change of food. This nurses' chart seems to us to be a very good one, and likely to be of great utility.

